# Hepatic and Nephric NRF2 Pathway Up-Regulation, an Early Antioxidant Response, in Acute Arsenic-Exposed Mice

**DOI:** 10.3390/ijerph121012628

**Published:** 2015-10-12

**Authors:** Jinlong Li, Xiaoxu Duan, Dandan Dong, Yang Zhang, Wei Li, Lu Zhao, Huifang Nie, Guifan Sun, Bing Li

**Affiliations:** 1 Department of Occupational and Environmental Health, School of Public Health, China Medical University, Shenyang 110013, China; E-Mails: lijinlong2201751@163.com (J.L.); duanxiaoxu1987@sina.com (X.D.); woddd1986@126.com (D.D.); zhangyang521516@163.com (Y.Z.); liwei09070710@163.com (W.L.); m13072474706@163.com (L.Z.); nhflzm@163.com (H.N.); 2 Cao County Center for Disease Control and Prevention, Heze 274400, China; 3 Environment and Non-Communicable Diseases Research Center, School of Public Health, China Medical University, Shenyang 110013, China; E-Mail: sungf@mail.cmu.edu.cn

**Keywords:** arsenic, ROS, NRF2, liver, kidney

## Abstract

Inorganic arsenic (iAs), a proven human carcinogen, damages biological systems through multiple mechanisms, one of them being reactive oxygen species (ROS) production. NRF2 is a redox-sensitive transcription factor that positively regulates the genes of encoding antioxidant and detoxification enzymes to neutralize ROS. Although NRF2 pathway activation by iAs has been reported in various cell types, however, the experimental data in vivo are very limited and not fully elucidated in humans. The present investigation aimed to explore the hepatic and nephric NRF2 pathway upregulation in acute arsenic-exposed mice *in vivo*. Our results showed 10 mg/kg NaAsO_2_ elevated the NRF2 protein and increased the transcription of *Nrf2* mRNA, as well as up-regulated NRF2 downstream targets HO-1, GST and GCLC time- and dose-dependently both in the liver and kidney. Acute NaAsO_2_ exposure also resulted in obvious imbalance of oxidative redox status represented by the increase of GSH and MDA, and the decrease of T-AOC. The present investigation reveals that hepatic and nephric NRF2 pathway expression is an early antioxidant defensive response upon iAs exposure. A better knowledge about the NRF2 pathway involvment in the cellular response against arsenic could help improve the strategies for reducing the cellular toxicity related to this metalloid.

## 1. Introduction

Arsenic is one of the most important natural pollutants worldwide [1]. Groundwater contaminated with inorganic arsenic (iAs) is the main source of human exposure and represents a global health issue [2,3]. Arsenic is an established human carcinogen and long-term exposure to iAs has been associated with cancers of the skin, lung, bladder, liver and kidney [4,5,6]. In addition to cancers, numerous epidemiological studies have also established a strong correlation between chronic iAs exposure and various human diseases such as hyperkeratosis, atherosclerosis, diabetes and chronic obstructive pulmonary diseases [7,8]. Arsenic damages biological systems through multiple mechanisms, one of them being reactive oxygen species (ROS) production [9,10]. The rapid induction of ROS has been observed in human bladder epithelial cells [11] and keratinocytes [12] exposed to arsenite and monomethylarsonous acid. It is further reported that arsenic could increase lipid peroxidation, deplete glutathione (GSH), and decrease the enzyme activities of superoxide dismutase (SOD), catalase (CAT), and glutathione peroxidase (GPx) both in the liver and kidney of rats [13,14,15].

Nuclear factor erythroid 2-related factor 2 (NRF2) is a redox-sensitive trascription factor that positively regulates the expression of genes encoding antioxidants, xenobiotic detoxification enzymes, and drug efflux pumps and confers cytoprotection against ROS and xenobiotics in normal cells [16]. Studies that effects of arsenic exposure on NRF2 signaling pathway were mostly conducted in cell culture models. Arsenite and arsenate are proved to induce the increase of NRF2 in osteoblasts, followed by transcriptional activation of target genes encoding *Hmox1*, *Prx1*, and one of a class of ubiquitin-binding proteins (A170) [17]. In human keratinocyte HaCaT cells, selective knockdown of *NRF2* by lentiviral short hairpin RNAs significantly reduce the expression of many antioxidant enzymes and sensitize the cells to acute cytotoxicity of arsenite [18]. What’s more, it is also demonstrated that compromised NRF2 expression sensitize the human bladder epithelial cells UROtsa to arsenite- and monomethylarsonous acid- induced toxicity by stably infected with NRF2-siRNA [19]. Aside from the cell lines mentioned above, at present, arsenicals also have been shown to activate the NRF2 pathway in placental choriocarcinoma cells [20], HeLa [21], myeloma cells [22], and breast cancer cells (MDA-MB-231) [23], indicating arsenic could induce the activation of NRF2 pathway in various cell types, and NRF2 pathway plays an extremely pivotal role in protection against arsenic-mediated toxicity *in vitro*.

The liver is the most important site of arsenic reduction and methylation [24]. Epidemiology studies have clearly indicated an association between chronic arsenic exposure and abnormal liver function, hepatomegaly, hepatoportal sclerosis, liver fibrosis and liver cancer [25]. The kidney, as the primary organ for the excretion of metabolites, is also suspected in recent years to be one of the main targets of arsenic [26,27]. The development of acute tubular necrosis with acute renal failure has been reported in patients with systemic toxicity occurring in severe acute arsenic poisoning, and some of these patients develop cortical necrosis and progression to chronic kidney disease (CKD) [28]. Hepatic NRF2 protein levels in Chang human hepatocytes increased rapidly and the endogenous NRF2-regulated downstream *Hmox1* mRNA and protein were induced dramatically after acute arsenite treatment [29]. After six weeks of arsenic exposure, *Nrf2^-/-^* mice displayed more severe pathological changes in the liver and hepatocytes were more sensitive to arsenic-induced DNA hypomethylation, oxidative DNA damage, and apoptotic cell death compared with *Nrf2^+/+^* mice [30]. Overall, as regards the animal experimental data of arsenic exposure on hepatic and nephritic NRF2 pathway *in vivo* are not sufficient, the potential meaning and possible mechanisms of NRF2 pathway activation by arsenic remain to be further elucidated. In the present study, mice were exposed to environmentally relevant concentrations of iAs intra-gastrically, we observed changes of hepatic and nephric NRF2 pathway *in vivo*, we also confirmed the imbalance of hepatic and nephric redox status in mice. 

## 2. Materials and Methods

### 2.1. Reagents and Chemicals

Sodium arsenite (NaAsO_2_, ≥99.0%) was obtained from Sigma Chemical Co. (St. Louis, MO, USA). NaAsO_2_ was dissolved in distilled water and diluted to the desired concentrations. Glutathione (GSH), maleic dialdehyde (MDA) and total antioxidative capacity (T-AOC) assay kits were purchased from Jiancheng Biological Institute (Nanjing, China). Primary antibodies of NRF2 (H-300: sc-13032), HO-1 (H-105: sc-10789), GSTO1/2 (FL-241: sc-98560), GCLC (H-300: sc-28965), β-actin (I-19: sc-1616) and corresponding secondary antibodies were all purchased from Santa Cruz Biotechnology (Santa Cruz, CA, USA). Real-time polymerase chain reaction (real-time PCR) kits were from Takara Co. (Otsu, Japan). Potassium hydroxide (KOH), hydrochloric acid (HCl) and potassium borohydride (KBH_4_) were purchased from Shanghai Chemical Co. (Shanghai, China) with arsenic free (<0.01 mg/L). All other chemicals used were of the highest grade commercially available. Water used in all the preparations was distilled and deionized.

### 2.2. Animals and Experimental Procedures

Female Kunming mice (weighing 18–22 g, 6–7 weeks old) were obtained from the Center for Experimental Animals at China Medical University (Shenyang, China) with a National Animal Use License number of SCXK-LN2011-0009. Animal use has been approved by Animal Use and Care Committee at China Medical University with a protocol number of CMU62043006. All experiments and surgical procedures were approved by the Animal Care and Use Committee at China Medical University, which complies with the National Institutes of Health Guide for the Care and Use of Laboratory Animals. All efforts were made to minimize the number of animals used and their suffering.

Mice were group-housed in stainless steel cages (6 mice per cage) in an air-conditioned room with temperature maintained at 20 ± 2 °C and 12/12 h light/dark cycle and year round relative humidity of 50%–60% for 1 week before the experiment. The mice were allowed standard mice chow diet and drinking water ad libitum throughout the study. The doses of NaAsO_2_ were selected on the basis of previously published studies [31,32] as well as our preliminary experiments. Mice were exposed to environmentally relevant concentrations of NaAsO_2_ (5, 10 and 20 mg/kg) intragastrically for 6, 12, 24, 48 and 72 h, respectively. Control mice were treated with saline only. At each end point of the treatment, all mice were weighed and killed by ether anesthesia. The entire liver and kidney of control and acute NaAsO_2_-exposed mice were promptly removed and weighed, and then stored at −80 °C for future use.

### 2.3. Calculation of the Liver and Kidney Indexes

The liver and kidney indexes were calculated according to the formulas: (liver or kidney weight/body weight) × 100%, respectively [33].

### 2.4. Determination of Tissue Arsenic Levels in Liver and Kidney

The liver and kidney of experimental mice were washed with normal saline to remove blood and clots, and then homogenized on ice with 500 μL deionized water and 0.05 g tissues. Determination of arsenic species, As^V^, As^III^, monomethylarsonic acid (MMA) and dimethylarsinic acid (DMA) in liver and kidney was performed by a high-performance liquid chromatography-hydride generation-atomic fluorescence spectrometer (HPLC-HG-AFS, SA-10 Atomic Fluorescence Species Analyzer, Titan Co., Beijing, China), consisting of a liquid chromatographic column, a hydride generation equipment, and an atomic fluoresce detector [34]. Total arsenic (T-As) levels in liver and kidney were then calculated by summing up the levels of As^V^, As^III^, MMA and DMA. All samples were analyzed thrice, and the results were expressed as mean ± SD (n = 3).

### 2.5. Western Blot Analysis

To extract the total proteins of liver and kidney, liver and kidney tissues were homogenized in lysis buffer containing 20 mmol/L Tris-HCl (pH = 8.0), 150 mmol/L sodium chloride, 1 mmol/L ethylene diaminetetraacetic acid, 1 mmol/L ethylene glycol tetraacetic acid, 0.5% nonidet P-40 (NP-40), 2.5 mmol/L sodium pyrophosphate, 1 mmol/L sodium orthovanadate and 1% protease inhibitor cocktail (Roche, Germany). Total protein concentrations were measured by Bradford’s method [35] using bovine serum albumin (Santa Cruz, CA, USA) as the protein standard. Aliquots of supernatant (40 μg total protein) were boiled in an equal volume of 2 × SDS electrophoresis sample buffer (0.2 mol/L Tris-HCl, 10% glycerol, 2% sodium dodecyl sulfate and 0.02% β-mercaptoethanol) for 5 min before 7.5%–10% sodium dodecyl sulfate-polyacrylamide gel electrophoresis (SDS-PAGE). After electrophoresis, proteins were transferred to polyvinylidene flfluorid (PVDF) membrane (Amersham, Buckinghamshire, UK). The blots were placed in blocking solutions (PBS containing 80 mmol/L disodium hydrogen phosphate, 25 mmol/L sodium dihydrogen phosphate, 100 mmol/L sodium chloride, 0.1% Tween 20 and 5% skim milk) for 2 h at room temperature. Blots were then incubated with the primary antibodies of NRF2, HO-1, GSTO1/2 and GCLC at 4 °C overnight, respectively. On the second day, membranes were washed and incubated with corresponding secondary antibodies (1:1000–5000 dilution) for 2 h at room temperature. Blots were then incubated with chemiluminescence reagents (PicoWest Super Signal, Pierce Biotechnology, Rockford, IL, USA) and visualized using Electrophoresis Gel Imaging Analysis System (MF-ChemiBIS 3.2, DNR Bio-Imaging Systems, Jerusalem, Israel). β-actin (1:5000) was used as the internal control. Representative bands from three mice of each treatment group were presented in the results.

### 2.6. Total RNA Isolation and Real-Time PCR Analysis

Total RNA of liver and kidney from experimental mice was isolated using a Trizol Reagent (Invitrogen, Carlsbad, CA, USA,). Real-time PCR was conducted using a two-step method with an ABI 7500 Real-Time PCR System (ABI, Carlsband, CA, USA). Briefly, 500 ng of total RNA was reverse-transcripted (RT) to cDNA using PrimeScript RT reagent kit with gDNA eraser (Perfect Real Time, Takara, Otsu, Japan), and PCR amplification was performed by SYBR Premix Ex Taq II kit (Perfect Real Time, Takara). PCR amplification conditions were: 1 cycle of initial denaturation (95 °C for 30 s), and 40 cycles of amplification (95 °C for 5 s and 60 °C for 34 s). Primers for mouse genes were designed by PRIMER 3 software and synthesized by Sangon Biological Engineering Technology (Shanghai, China) as follows: *Nrf2* accession number (NM_010902.3): forward (ttggcagagacattcccatttg) and reverse (aaacttgctccatgtcctgctcta); *Hmox1* accession number (NM_010442): forward (tgcaggtgatgctgacagagg) and reverse (gggatgagctagtgctgatctgg); *Gsto1* accession number (NM_010362.2): forward (cttcatggcgtagttgaatgatgtc) and reverse (tttaagtactcgcggtaggtcttgg); *Gclc* accession number (NM_010295): forward (cagtcaaggaccggcacaag) and reverse (caagaacatcgcctccattcag); *Gapdh* accession number (NM_001289726): forward (tgtgtccgtcgtggatctga) and reverse (ttgctgttgaagtcgcaggag). All primer sets were tested prior to use in this work to ensure that only a single product of the correct size was amplified. Triplicate reactions were performed for each sample. Cycle threshold (Ct) values were obtained graphically for both different target genes and *Gapdh*. The Ct values of different target genes were first normalized to *Gapdh* in the same sample and expressed as ΔCt values. Then ΔΔCt values were obtained by subtracting the ΔCt values of the control samples from that of the treated samples, and 2^−ΔΔCt^ values were calculated to represent the amounts of different target genes. The final values presented were expressed as folds of control. 

### 2.7. Analysis of GSH Levels in the Liver and Kidney

GSH levels were determined by modified 5,5’-dithiobis 2-nitrobenzoic acid (DTNB) method [36] using commercially available kit according to the manufacturer’s recommended protocol. The liver and kidney from each mice was washed with normal saline to remove blood and clots, and then homogenized on ice with 5 ml 5% trichloroacetic acid (TCA) per gram of tissue weight. Homogenates were centrifuged at 1000 g for 15 min at 4 °C and the aliquots samples of the supernatants were then used for the analysis of liver and kidney GSH. The levels of GSH in liver and kidney were finally expressed as μg/100 mg tissue.

### 2.8. Analysis of Lipid Peroxidation and T-AOC in the Liver and Kidney

The liver and kidney of experimental mice were washed with normal saline to remove blood and clots, and then homogenized on ice with 9 ml Tris-HCl (5 mmol/L containing 2 mmol/L ethylene- diaminetetraacetic acid, pH = 7.4) per gram of tissue weight. Homogenates were then centrifuged at 1000 × g for 15 min at 4 °C and the supernatants were used for the analysis of lipid peroxidation and T-AOC in liver and kidney according to each manufacturer’s recommended protocol. Lipid peroxidation was determined by measuring the levels of MDA, an end product of lipid peroxidation, using a thiobarbiturate method [37]. The levels of MDA were fi The expressed as nmol/mg protein. T-AOC levels were determined basing on the color changes of tetramethylbenzidine (TMB) spectrophotometrically at 405 nm [38]. The unit of T-AOC was defined as each 0.01 increasement of TMB optical density (OD) per minute at 37 °C, and T-AOC levels were finally expressed as U/mg protein. 

### 2.9. Statistical Analysis

A statistician was consulted before the start of the experiment for the minimum number of mice required to give viable statistical and reproducible data and for statistical analysis. Data were presented as mean ± SD. Statistical significance was determined by one-way analysis of variation (ANOVA) followed by post hoc analysis using Least-Significant Difference (LSD) method (SPSS 11.0, SPSS Inc., Chicago, IL, USA). *p* values of less than 0.05 were considered as statistically significant.

## 3. Results

### 3.1. T-As Concentrations, as Well as the Indexes of Liver and Kidney in Control and Acute Arsenic-Exposed Mice

In our study, mice were treated intragastrically with 0, 5, 10 and 20 mg/kg of NaAsO_2_. We detected T-As concentrations at different time points and found that the peaks of T-As concentrations were at 6 h in the liver and at 1 h in the kidney, then T-As levels decreased gradually (data not shown). There were significantly dose-dependent trends of T-As levels both in the liver and kidney with indicated time points (Table 1). It was therefore quite clear that NaAsO_2_ had been quickly absorbed into blood and distributed to the liver and kidney. In addition, we calculated the indexes of liver and kidney in each experimental group and didn’t find any significant difference (Table 1), demonstrating no obvious hepatic and nephric swelling were induced with indicated arsenic doses.

**Table 1:** Total arsenic levels (T-As, ng As/g tissue), as well as the indexes of liver and kidney in control and acute arsenic-exposed mice.

NaAsO_2_ (mg/kg)	T-As in Liver	T-As in Kidney	Liver Index (%)	Kidney Index (%)
0	< LD	< LD	5.36 ± 0.39	1.21 ± 0.07
5	359.86 ± 28.59	1165 ± 303.00	5.05 ± 0.34	1.17 ± 0.14
10	1033.09 ± 106.66 *	1816 ± 279.8 *	4.49 ± 0.20	1.14 ± 0.10
20	3075.36 ± 485.11 *^,#^	2332 ± 174.60 *	5.22 ± 0.28	1.27 ± 0.10

Notes: mice were treated with 0, 5, 10 and 20 mg/kg NaAsO_2_ intragastrically, and total arsenic (T-As) levels of the liver (6 h) and kidney (1 h) were determined respectively by the HPLC-HG-AFS method, as described in materials and methods. Results were expressed as mean ± SD (n = 3). The entire liver and kidney were removed and weighed after acute arsenic-treatment for 24 h, and liver and kidney indexes were expressed as liver or kidney weight/body weight (n = 10). The limit of detection (LD) for T-As was 1 μg/L. * denoted *p* < 0.05 compared with 5 mg/kg NaAsO_2_ treatment group. ^#^ denoted *p* < 0.05 compared with 10 mg/kg NaAsO_2_ treatment group.

### 3.2. Up-Regulation of Nuclear Factor NRF2 both in the Liver and Kidney of Control and Acute Arsenic-Exposed Mice

**Figure 1:**
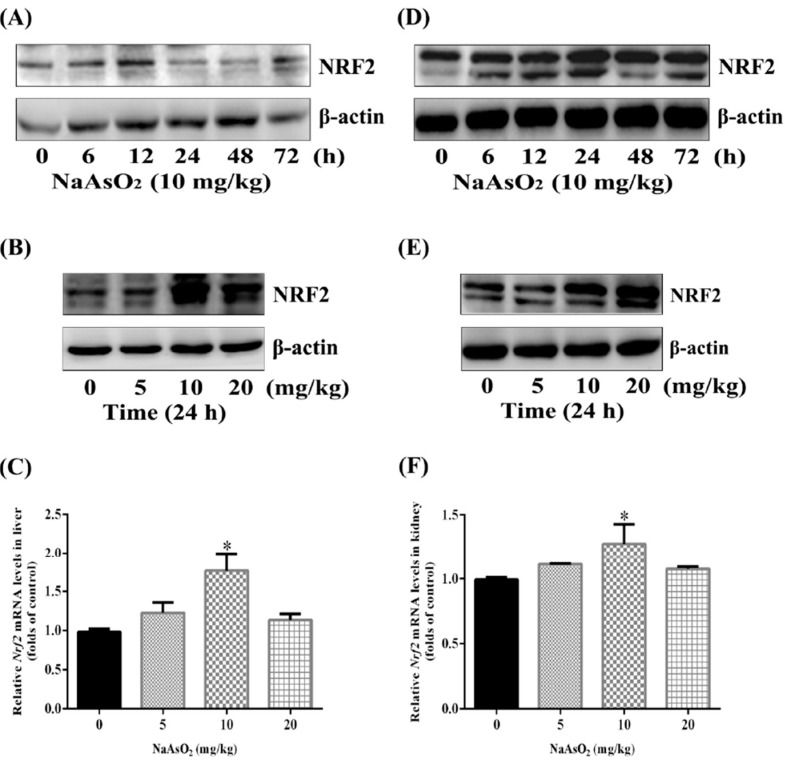
Up-regulation of NRF2 both in the liver and kidney of control and acute arsenic-exposed mice. Mice were treated with 0, 5, 10 and 20 mg/kg NaAsO_2_ intra-gastrically for 6, 12, 24, 48 and 72 h. The extracted proteins of liver and kidney were subjected to SDS-PAGE. Expression of NRF2 protein in the liver (**A, B**) and kidney (**D, E**) of mice were assessed by western blotting. β-Actin was blotted as the loading control. Total RNA of liver and kidney from each 6 h experimental group were isolated and real-time PCR were conducted. The mRNA levels of *Nrf2* in the liver and kidney were shown as (**C**) and (**F**). Results were expressed as mean ± SD (n = 4), and independent experiments were carried out three times. * denotes *p* < 0.05 compared with control mice.

Several studies have reported that Nrf2 regulates critically cellular defense transcriptional programs against iAs toxicity to maintain cellular redox homeostasis and limit oxidative damages *in vitro* [18,19]. In order to explore whether acute arsenic exposure could induce NRF2 pathway *in vivo*, we first treated the mice intragastrically with 10 mg/kg NaAsO_2_ for 6, 12, 24, 48 and 72 h, and proved the elevation of NRF2 protein both in the liver and kidney. However, the elevation of NRF2 protein in the liver was detectable as soon as 6 h and peaked at 12 h (Figure 1A), while the activation of NRF2 in the kidney was more durable and lasted to as long as 72 h (Figure 1D). We next confirmed the expression of NRF2 protein both under 10 and 20 mg/kg NaAsO_2_ *in vivo* (Figure 1B,E). What’s more, the transcription of *Nrf2* mRNA in the liver and kidney was also moderately increased by 5, 10 and 20 mg/kg NaAsO_2_ treatment for 6 h compared with control group (Figure 1C,F). 

### 3.3. Up-Regulation of NRF2 Downstream Targets both in the Liver and Kidney of Control and Acute Arsenic-Exposed Mice 

**Figure 2:**
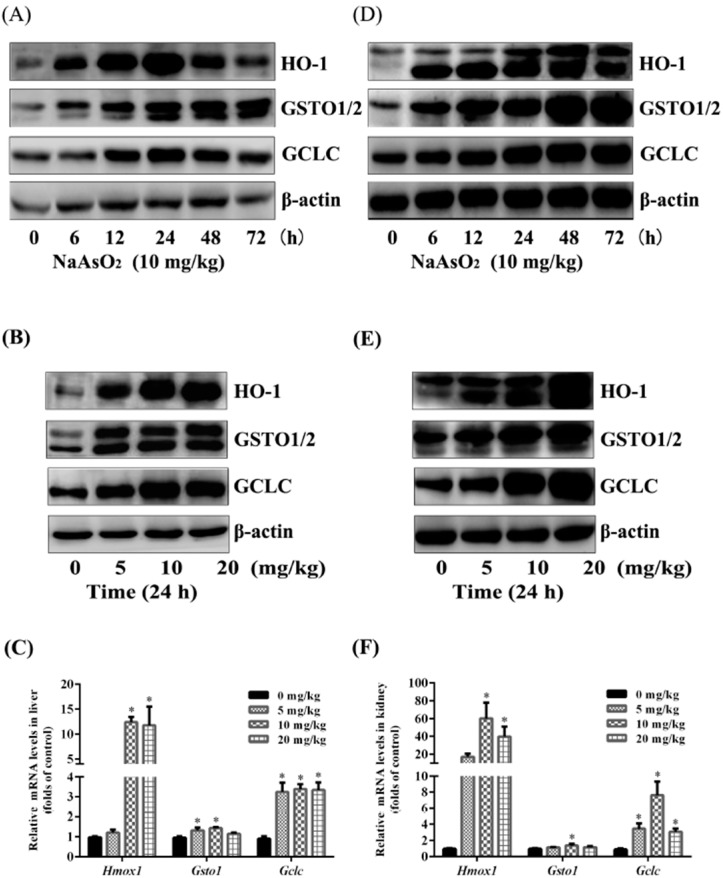
Up-regulation of NRF2 downstream targets both in the liver and kidney of control and acute arsenic-exposed mice. Mice were treated with 0, 5, 10 and 20 mg/kg NaAsO_2_ intra-gastrically for the 6, 12, 24, 48 and 72 h, and the extracted proteins of liver and kidney were subjected to SDS-PAGE. Expressions of HO-1, GSTO1/2 and GCLC in the liver (**A, B**) and kidney (**D, E**) of mice were assessed by western blotting. β-Actin was blotted as the loading control. Total RNA of liver and kidney from each 6 h experimental group was isolated and real-time PCR were conducted. The mRNA levels of *Hmox1*, *Gsto1 and Gclc* in liver and kidney were shown as (**C**) and (**F**). Results were expressed as mean ± SD (n = 4), and independent experiments were carried out three times. * denotes *p* < 0.05 compared with control mice.

The antioxidant enzyme HO-1, phase II drug-metabolizing enzyme GST, and the rate-limiting enzyme of GSH biosynthesis GCL are all typically recognized NRF2 downstream targets, these enzymes and proteins are responsible for the detoxication of electrophiles and ROS, as well as the removal or repair of some of their damage products. Our results here also showed that NaAsO_2_ could up-regulated hepatic and nephric HO-1, GST and GCLC protein expression time-dependently (Figure 2A,D) and dose-dependently (Figure 2B,E). In addition, the transcription of *Gsto1* and *Gclc* mRNA was raised remarkly by 5, 10 and 20 mg/kg NaAsO_2_ treatment for 6 h, particularly, the *Hmox1* levels increased dramatically, which was in agreement with previous reports in many cell types and tissues (Figure 2C,F) [29,30]. Totally, these results confirmed the quick activation of hepatic and nephric NRF2 pathway by acute arsenic exposure *in vivo*. 

### 3.4. Acute NaAsO_2_ Exposure Results in Oxidative Stress both in the Liver and Kidney

**Figure 3:**
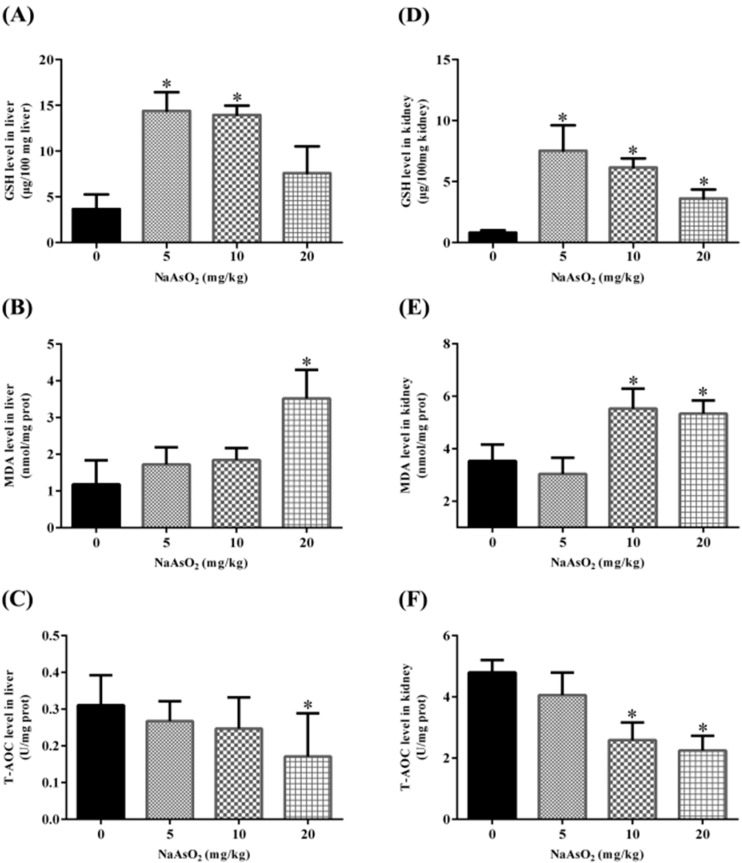
Effects of acute NaAsO_2_ exposure on GSH, MDA and T-AOC levels in the liver and kidney of control and acute arsenic-exposed mice. Mice were treated with NaAsO_2_ intra-gastrically for 24 h with indicated doses. The levels of GSH, MDA and T-AOC in the liver (**A**–**C**) and kidney (**D**–**F**) were all determined using commercially available kits as described in materials and methods. Data were presented with means ± SD (n = 10). * denotes *p* < 0.05 compared with control mice.

Oxidative stress is the most widely accepted and studied mechanism of arsenic toxicity [39]. Oxidative stress represents an imbalance between the production and manifestation of ROS and a biological system’s ability to readily detoxify the reactive intermediates or to repair the resulting damage. In this study, we detected the oxidative stress related markers in the liver and kidney upon acute arsenic exposure for 24 h. GSH, one of the most important antioxidants to defend against ROS by xenobiotics, is an abundant tripeptide widely distributed in various of tissues [40], and increased notably both in the liver (Figure 3A) and kidney (Figure 3D), suggesting the imbalance of body oxidative redox status. What’s more, the increase of MDA, an end production of lipid peroxidation [41,42], together with the decrease of T-AOC (reflecting body total antioxidant capacity) both in the liver (Figure 3B,C) and kidney (Figure 3E,F), revealed jointly the obvious oxidative stress by acute oral administration of iAs. 

## 4. Discussion

Arsenic is well known to cause numerous acute and chronic adverse health effects [5,6]. Although the mechanism of arsenic-induced carcinogenesis remains to be investigated, mechanistic studies have also confirmed that exposure to various arsenic compounds results in ROS generation [43,44], and excessive generation of intracellular ROS may lead to oxidative stress, loss of cell function, and ultimately apoptosis or necrosis [45]. Recent years, the critical roles of nuclear factor NRF2 in defending against elevated ROS and toxic damages by initiating the expressions of a variety of antioxidant enzymes and phase II drug-metabolizing enzymes have attracted great interest [46,47]. In this manuscript, we investigated the quick up-regulation of hepatic and nephric NRF2 pathway and the imbalance of oxidative redox status both in the control and acute arsenic-exposed mice *in vivo*. 

Vertebrates have evolved several defense mechanisms to cope with environmental insults and to maintain cellular redox homeostasis. As a key regulator of the antioxidant system, transcription factor NRF2 exists in cytoplasm with its repressor keap1, and NRF2 will disassociate with keap1 and enter into the nuclear to combine with antioxidant response element ARE when stimulated by xenobiotics to induce the expression of a set of phase II drug-metabolizing and antioxidant enzymes [48]. Activation of the NRF2 pathway has been clearly demonstrated to confer protection against toxic and carcinogenic effects of many environmental insults [49,50]. In accordance with our findings here, the quick up-regulation of NRF2 pathway and the imbalance of oxidative redox status by acute NaAsO_2_ exposure were demonstrated in HepG2 cells [51]. Our previous study also reported the dose-effect response of NRF2 accumulation as well as activation of an array of NRF2 downstream target genes by inorganic arsenic in a cultured human hepatocyte line [52]. As to the molecular mechanism of Nrf2 activation by arsenic, it is demonstrated that As^III^ is able to activate Nrf2 by increasing association between Keap1 and Cul3, therefore disrupting the dynamic assembly/disassembly process of the Keap1-Cul3 E3 ubiquitin ligase complex. Reduced E3 ubiquitin ligase activity inhibite Nrf2 ubiquitination and degradation, therefore enhance Nrf2 protein levels [23]. In addition, we found that acute arsenic exposure moderately increased hepatic and nephric *Nrf2* mRNA levels to some extent in our experiments, which is also confirmed by other studies [52,53]. It is therefore suggested that multiple mechanisms might be involved in Nrf2 activation, Which remain to be further studied.

It is reported that HO-1 degrades pro-oxidant heme into ferrous iron, carbon monoxide, and biliverdin which is quickly converted into bilirubin. The end-products of HO-1 have antioxidant activities that are able to defend cells from oxidative stress [52]. As one of phase II drug-metabolizing enzymes, GST plays an irreplaceable role in catalyzing GSH with its substrate to get rid of hazardous substances [54,55]. The rate-limiting step in GSH biosynthesis is catalyzed by glutamate cysteine ligase (GCL), a heterodimer composed of catalytic subunit GCLC and modifier subunit GCLM. The GCLC possesses all of the catalytic activity, while the GCLM functions to increase the catalytic efficiency of GCLC [56,57]. In our study, the dose- and time-dependent up-regulation of NRF2 downstream regulated targets HO-1, GST and GCLC protein expression both in the liver and kidney that they are thought to elicit beneficial effects and to play defensive roles in resisting the toxicity and redox imbalances of inorganic arsenic. 

The NRF2-mediated early antioxidant response by toxic arsenic could be explained by the phenomena called adaptive response or hormesis, that is, preconditioning cells with sublethal doses of toxic compounds increases cellular resistance to similar types of toxic compounds [58]. Many researchers nowadays have speculated that the activation of the ARE-NRF2-keap1 pathway by arsenic is beneficial and is likely to be an attempt of cells to counteract the damage effects of the metalloid [59]. However, the protective mechanism of the NRF2 pathway may be masked by cell damaging effects at high concentrations of arsenic, and the NRF2-dependent defense response is outweighed by the deteriorated effects induced by arsenic, ultimately, resulting in oxidative stress and toxicity, which could also be shown in our results that the hepatic and nephric imbalance of oxidative redox status represented by the increase of GSH and MDA, as well as the decrease of T-AOC both in control and acute arsenic-exposed mice, in accordance with previous study [60,61,62]. 

## 5. Conclusions

Collectively, our findings here showed the hepatic and nephric early adaptive response derived from activation of the NRF2 pathway by iAs *in vivo*, which represents the initial attempt to counteract the deteriorated effects and to maintain cellular homeostasis when exposure to the toxic metalloid. It is therefore indicated the identification, validation, and optimization of new Nrf2 activators might be extremely essential for potential roles of dietary and therapeutic interventions to boost the Nrf2-dependent adaptive system to resist against arsenic adverse effects.
